# Gait-Adaptability Training in People With Hereditary Spastic Paraplegia: A Randomized Clinical Trial

**DOI:** 10.1177/15459683221147839

**Published:** 2023-01-25

**Authors:** Lotte van de Venis, Bart van de Warrenburg, Vivian Weerdesteyn, Alexander C. H. Geurts, Jorik Nonnekes

**Affiliations:** 1 Department of Rehabilitation, Center of Expertise for Rare and Genetic Movement Disorders, Donders Institute for Brain, Cognition and Behavior, Radboud University Medical Center, Nijmegen, The Netherlands; 2 Department of Neurology, Center of Expertise for Rare and Genetic Movement Disorders, Donders Institute for Brain, Cognition and Behavior, Radboud University Medical Center, Nijmegen, The Netherlands; 3 Research, Sint Maartenskliniek, Nijmegen, The Netherlands; 4 Department of Rehabilitation, Sint Maartenskliniek, Nijmegen, The Netherlands

**Keywords:** Hereditary spastic paraplegia, C-Mill, gait adaptability, rehabilitation, physical therapy

## Abstract

**Background and objectives:**

In people with hereditary spastic paraplegia (HSP), reduced gait adaptability is common and disabling. Gait impairments result from lower extremity spasticity, muscle weakness, and impaired proprioception. The aim of this study was to assess the efficacy of a 5-week gait-adaptability training in people with pure HSP.

**Method:**

We conducted a randomized clinical trial with a cross-over design for the control group, and a 15-week follow-up period after training. Thirty-six people with pure HSP were randomized to 5 weeks of (i) gait-adaptability training (10 hours of C-Mill training—a treadmill equipped with augmented reality) or (ii) a waiting-list control period followed by 5 weeks gait-adaptability training. Both groups continued to receive usual care. The primary outcome was the obstacle subtask of the Emory Functional Ambulation Profile. Secondary outcome measures consisted of clinical balance and gait assessments, fall rates, and spatiotemporal gait parameters assessed via 3D motion analysis.

**Results:**

The gait-adaptability training group (n = 18) did not significantly decrease the time required to perform the obstacle subtask compared to the waiting-list control group (n = 18) after adjusting for baseline differences (mean: −0.33 seconds, 95% CI: −1.3, 0.6). Similar, non-significant results were found for most secondary outcomes. After merging both groups (n = 36), the required time to perform the obstacle subtask significantly decreased by 1.3 seconds (95% CI: −2.1, −0.4) directly following 5 weeks of gait-adaptability training, and this effect was retained at the 15-week follow-up.

**Conclusions:**

We found insufficient evidence to conclude that 5 weeks of gait-adaptability training leads to greater improvement of gait adaptability in people with pure HSP.

## Introduction

Hereditary spastic paraplegia (HSP) is a heterogenous group of neurodegenerative disorders. It is caused by retrograde axonal degeneration of the corticospinal tract, posterior spinal columns, and spinocerebellar fibers.^1,2^ Pure forms of HSP are clinically characterized by progressive bilateral spasticity, muscle weakness, and a reduced proprioception of the lower extremities.^1,2^ These symptoms result in disabling gait and balance impairments, including difficulties adapting the walking pattern to meet environmental demands (e.g., stepping over an obstacle or speeding up walking to cross the street). This hinders safe and independent ambulation in the community.^3,4^

Previous uncontrolled studies with pre-post assessments reported beneficial effects on balance and/or gait performance following task-specific gait training in people with HSP. The interventions consisted of 18 sessions of robotic Lokomat training (n = 13),^5^ a combined intervention of botulinum toxin type-A injections followed by 10 sessions of physical therapy (n = 18),^6^ 25 sessions of robot-assisted exoskeleton and overground walking exercises (n = 1),^7^ or a low-intensity 12-week physical therapy program (n = 1).^8^ Although the results of these studies are promising, none of these studies included outcome measures aimed at evaluating gait adaptability, nor did the applied gait training interventions include context-specific tasks that specifically targeted gait adaptability. Context-specific gait-adaptability training can be provided on the C-Mill, a treadmill equipped with augmented reality. Via visual projections on the treadmill, participants can train several domains of gait adaptability (e.g., obstacle negotiation and precision stepping) in a safe environment. Previous studies have demonstrated feasibility and efficacy of gait-adaptability training on the C-Mill in chronic stroke patients,^9-11^ people with cerebellar degeneration,^12^ and persons with Parkinson’s disease.^13^

So far, the potential effectiveness of gait-adaptability training has not been studied in people with HSP. Therefore, we designed and conducted the Move-HSP trial^14^: a randomized clinical trial to compare the effect of gait-adaptability training added to usual care, with usual care alone. We hypothesized that the addition of gait-adaptability training would result in greater improvements of gait-adaptability performance as evaluated with the obstacle subtask of the Emory Functional Ambulation Profile (E-FAP).^15^ In addition, clinical balance and gait measures, balance confidence, spatiotemporal gait parameters, and level of physical activities in daily life were evaluated, and hypothesized to improve more by the addition of gait-adaptability training.

## Methods

### Study Design and Setting

We conducted a 5-week, randomized clinical trial, with a cross-over design for the waiting-list control group, and a 15-week follow-up period after the intervention. This mono-center study was conducted at the Center of Expertise for Rare and Genetic Movement Disorders of the Radboud University Medical Center (Nijmegen, The Netherlands), which is part of the European Reference Network for Rare Neurological Diseases (ERN-RND). Ethical approval was obtained from the Medical Ethical Committee Oost-Nederland, the Netherlands (2019-5602, NL70295.091.19). The trial was registered on Clinicaltrials.gov (NCT04180098) and the study protocol has previously been published.^14^ All participants signed informed consent. Participants were randomized via a web-based randomization system into 2 groups: (i) *gait-adaptability training group*: 5 weeks of C-Mill training next to usual care; or (ii) *waiting-list control group* receiving 5 weeks of usual care. Randomization was done following a 1:1 ratio with randomly varying blocks (n = 4 or n = 6) and stratified by disease duration (2 categories: 0-15 years and >15 years). Participants in the gait-adaptability training group were assessed 3 times; pre training, post training, and at follow-up. Participants assigned to the waiting-list control group crossed over to 5 weeks of gait-adaptability training following 5 weeks of usual care. Therefore, participants in the waiting-list control group were assessed 4 times: pre waiting list, pre training, post training, and at follow-up. A detailed flowchart of the study design is available in the previously published protocol paper.^14^ The assessments took place at the movement laboratory of the Radboud University Medical Center (Nijmegen, The Netherlands). The gait-adaptability training sessions were executed at 4 sites: Radboud University Medical Center (Nijmegen, NL), Paramedisch Centrum Rembrandt (Veenendaal, NL), Stichting Tante Louise (Bergen op Zoom, NL), and Fysiotherapie De Lindehoeve (Voorschoten, NL). Participants could not be blinded, as they unavoidably knew whether they received gait-adaptability training or not. The assessor (LV) conducting the measurements at the movement laboratory also provided the training sessions at Radboud University Medical Center and could, therefore, not be blinded either. During the trial, all participants were allowed to continue with usual care (e.g., physical therapy).

### Participants

Participants were recruited via the outpatient clinic of the Center of Expertise for Rare and Genetic Movement Disorders of the Radboud University Medical Center and via the Dutch HSP working group of the patient organization “Spierziekten Nederland.” Inclusion criteria were (1) diagnosis of pure HSP by a neurologist specialized in genetic movement disorders, (2) aged between 18 and 70 years old, and (3) ability to walk barefoot on a level ground without a walking aid (use of orthopedic devices was allowed). Participants were excluded if they suffered from concomitant neurological, orthopedic or psychiatric conditions that might affect gait performance, or if they had any HSP-related surgical procedure of the lower extremities in their medical history. Participants provided demographic information including age and sex. Clinical characteristics that were recorded consisted of leg muscle tone assessed with the Modified Ashworth Scale (MAS; range 0-5),^16^ leg muscle strength assessed with the Medical Research Council scale (MRC; range 0-5),^17^ vibration sense at the ankles and feet assessed with a semiquantitative tuning fork (Rydel-Seiffer, Neurologicals Poulsbo, Washington) (range 0-8). In addition, we recorded disease duration (years), level of disease severity assessed with the Spastic Paraplegia Rating Scale (SPRS; range 0-52),^18^ trunk control assessed with the Trunk Control Measurement Scale (TCMS; range 0-58),^19^ and self-reported falls during the previous year.

### Intervention

Participants trained their gait adaptability on the C-Mill (Motek Medical, Culemborg, The Netherlands), a treadmill equipped with augmented reality. Visual projections onto the treadmill acted as stepping targets or obstacles to elicit step adjustments. The training sessions were guided by a physical therapist. Sessions lasted 60 minutes and took place twice a week for a period of 5 weeks, adding up to a total of 10 hours of gait-adaptability training. A detailed description of the training has previously been published.^14^ In short, training sessions started with a 10-minute warming-up, followed by 5 exercises of approximately 8 minutes. Each exercise focused on a specific gait-adaptability task: (A) precision stepping, (B) obstacle negotiation, (C) direction of progression, (D), precision acceleration, or (E) walking velocity. Sessions ended with a 5-minute game that combined several gait-adaptability tasks and a 5-minute cooling-down period. During the training sessions, additional short periods of rest were provided as needed. To ensure sufficient challenge for each participant, progression of task complexity was moderated by the therapist based on the participants capacity. A maximum of 2 therapists per participant were involved in providing gait-adaptability training sessions.

### Outcome Measures

The primary outcome was gait adaptability assessed with the obstacle subtask of the E-FAP.^15^ Secondary outcomes consisted of the Mini Balance Evaluation Test (MiniBEST),^20^ Activities-specific Balance Confidence scale (ABC), the Walking Adaptability Ladder Test (WALT),^21^ and the 10-meter Walk Test (10 mWT).^22^ In addition, 3-dimensional gait analysis (Vicon© Motion Systems Ltd.) was performed. To this end, retroreflective markers were placed according to the standard Plug-In-Gait upper and lower body marker model. During the gait analysis, participants walked 2 bouts of 3 minutes at comfortable speed over an 8-m walkway with their own comfortable shoes. From the gait analysis, average stride length (m), average stride time (s), average step width (m), walking speed (m/s), and cadence (steps/min) were extracted. The assessments were conducted by a trained investigator using a standardized protocol. Details on how the outcome measures were assessed have been published in the study protocol.^14^ Use of orthotic devices, including orthopedic footwear, was allowed and kept consistent throughout the different assessments.

Furthermore, levels of physical activity were measured following each assessment during 7 consecutive days with activity monitors (Activ8, Remedy Distribution Ltd., Valkenswaard, The Netherlands). Physical activity was expressed as total time spent walking and total time spent active (i.e., minutes classified as walking, running, or cycling). Lastly, during 15 weeks prior to the first assessment and during 15 weeks following gait-adaptability training, all participants self-reported their falls and near-falls in a digital fall diary. Falls that occurred during these fall diary periods were considered to be outcomes. In contrast, falls that were registered during the 5 weeks gait-adaptability training or during the 5 weeks on the waiting-list were considered as adverse events.

### Sample Size

Sample size calculation is based on previous studies evaluating the effectiveness of C-Mill training on the obstacle subtask of the E-FAP (these studies involved stroke patients^9^ and people with ataxia^12^). To detect an improvement of 1.75 seconds on the E-FAP obstacle subtask (SD: 2.0) and applying an α = .05 and β = .2, 16 participants per group would be needed. Allowing a 10% attrition rate, we aimed for a total of 36 participants.^14^

### Statistical Analysis

The effects of gait-adaptability training on the primary and secondary outcome measures were assessed by comparing the post-intervention scores through analysis of covariance (ANCOVA). The baseline score of the corresponding primary and secondary outcomes was entered into the model as a covariate. Analyses were based on the intention-to-treat principle. In second instance, both groups were merged based on their corresponding pre-training, post-training, and follow-up assessments (i.e., assessments 1, 2, and 3 for the gait-adaptability training group and assessments 2, 3, and 4 for the waiting-list control group). Time effects during and after gait-adaptability training were then assessed with a repeated-measures analysis of variance (ANOVA) using time as a within-subjects factor. Post hoc testing with paired *t*-tests was done to assess whether outcomes differed between the post- versus pre-training assessments, and between the follow-up versus post-training assessments. The fall dairies were processed descriptively.

## Results

### Patient Enrollment, Adherence, and Adverse Events

**Figure 1.**
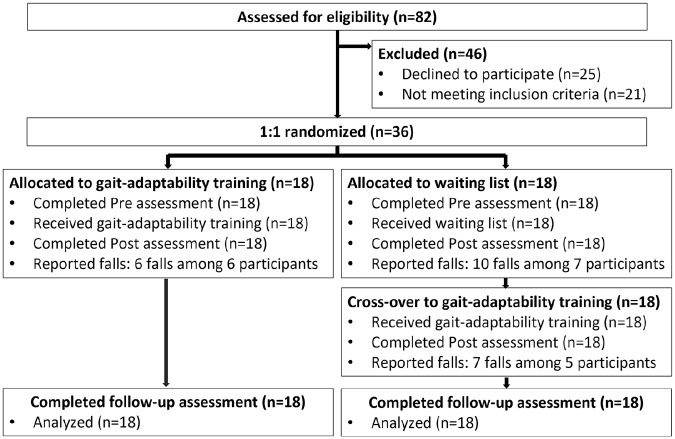
Flowchart of Move-HSP.

Participants were recruited between November 2019 and June 2021. Out of the 82 eligible people with pure HSP, 36 participants were enrolled and randomized (Figure 1). Eighteen participants were allocated to the gait-adaptability training group and 18 to the waiting-list control group. The overall adherence to the gait-adaptability training was 99.7% (359 out of 360 training sessions completed). There were no drop-outs during the study period. The disease severity of 2 participants in the gait-adaptability training group prevented them from performing the WALT. As a result, the WALT scores of 34 participants were included in the analysis. The completion rate of fall diaries was 97.2% (70 out of 72 fall diaries were returned completed). Therefore, the fall diaries of 2 participants were excluded from the analysis, as 1 participant did not complete the follow-up fall diary due to lack of motivation, and 1 participant experienced technical difficulties. As a result, the fall diaries of 34 participants were analyzed.

During the study period, 1 serious adverse event occurred. One participant touched electric wires when replacing a lamp during work. On advice of his general practitioner, this participant was admitted to hospital for 24 hours observation, after which he was discharged without residual symptoms. During the gait-adaptability training period, a total of 13 falls (range 1-2 per participant) were reported among 11 of the 34 participants; however, none of these falls occurred during the training itself. Lastly, during the 5-week waiting-list period, a total of ten falls (range 1-3 falls per participant) were reported among 7 out of 18 participants.

### Participant Characteristics and Co-interventions

The participants randomized to either the gait-adaptability training group or waiting-list control group did not differ in demographic or clinical characteristics (Table 1). A total of 16 participants received physical therapy as a co-intervention: in the gait-adaptability training group, 10 of the 18 participants received physical therapy for an average of 70 minutes per week (range 20-270); in the waiting-list control group, 6 of the 18 participants received physical therapy for an average of 100 minutes per week (range 20-180). No other co-interventions were reported.

**Table 1.** Clinical Characteristics of Participants per Group.

	Gait-adaptability training group	Waiting-list control group
Number of participants	18	18
Age (y)	47.6 (±8.8)	50.0 (±12.7)
Sex (men)	14	13
Time since first symptom (y)	15.8 (±13.5)	17.1 (±13.9)
Falls past year	3	5
Hip flexors		
MRC	4 [3-5]	4 [4-5]
Hip extensors		
MRC	4 [3-5]	4 [4-5]
Hip abductors		
MRC	5 [2-5]	4 [4-5]
Hip adductors		
MRC	5 [2.5-5]	4 [4-5]
MAS	1 [0-2.5]	1 [0-2.5]
Knee extensors		
MRC	4 [4-5]	4 [4-5]
MAS	1 [0-3]	0 [0-2.5]
Knee flexors		
MRC	4 [3-5]	4 [3-5]
MAS	1.25 [0-3]	1 [0-2.5]
Ankle plantarflexors		
MRC	5 [4-5]	5 [4-5]
MAS—knee extended	1.5 [0-3]	1 [0-2.5]
MAS—knee flexed	1 [0-3]	1.25 [0-2]
Ankle dorsiflexors		
MRC	5 [3.5-5]	4.5 [3-5]
MAS	0 [0-1]	0 [0-0.5]
Vibration sense—malleoli laterales	3.8 [0-8]	5.3 [0-8]
Vibration sense—head of metatarsal I	3.5 [0-8]	5.3 [0-8]
Spastic Paraplegia Rating Scale (range 0-52)	11.4 (±5.2)	10.1 (±3.6)
Trunk Control Measurement Scale (range 0-58)	46.3 (±5.7)	47.6 (±3.3)

Abbreviations: MRC, Medical Research Council; MAS, Modified Ashworth Scale.

Values displayed are means (±SD) or median [range]. MRC scores (0-5), MAS scores (0-5) and vibration sense scores (0-8) are averaged values of the left and right body side. Vibration sense was assessed using a semiquantitative tuning fork (Rydel-Seiffer, Neurologicals Poulsbo, Washington).

### Intervention Effects

The time required to perform the E-FAP obstacle subtask did not decrease more in the gait-adaptability training group compared to the waiting-list control group after adjustment for baseline differences (mean group differences: −0.33 seconds, 95% CI: −1.3, 0.6; *P* = .471). Similar non-significant results were found for the secondary outcomes, except for the single run of the WALT (mean group differences: −2.14 seconds; 95% CI: −4.1, −0.1; *P* = .037, see Table 2).

**Table 2.** Group Means Pre and Post Intervention Period and Mean Group Differences Post Intervention Period (ANCOVA).

	Gait adaptability training group (n = 18)			Waiting list control group (n = 18)			Gait adaptability training group vs waiting list control group	
	n	Pre (week 0)	Post (week 6)	n	Pre (week 0)	Post (week 6)	Mean group difference post intervention period adjusted for baseline values [95% CI]	*P* value
		Mean ± SD	Mean ± SD		Mean ± SD	Mean ± SD		
Clinical assessment								
Obstacle subtask E-FAP (s)	18	10.3 ± 6.6	8.6 ± 3.9	18	9.6 ± 4.7	8.5 ± 3.9	−0.33 [−1.3, 0.6]	.471
MiniBEST	18	18.3 ± 6.0	20.1 ± 5.4	18	19.3 ± 3.8	19.9 ± 3.8	1.09 [−0.4, 2.6]	.172
Activities-specific Balance Confidence Scale	18	66.5 ± 18.1	72.7 ± 16.2	18	70.9 ± 18.2	72.7 ± 16.6	3.40 [−2.7, 9.5]	.268
Ten-meter walk test—comfortabel (m/s)	18	1.3 ± 0.3	1.3 ± 0.3	18	1.2 ± 0.3	1.3 ± 0.3	0.03 [0.0, 0.1]	.387
Ten-meter walk test—fast (m/s)	18	1.6 ± 0.3	1.7 ± 0.4	18	1.7 ± 0.4	1.7 ± 0.3	0.04 [−0.1, 0.1]	.430
Walking Adaptability Ladder Test—single run (s)	16^A^	21.9 ± 13.8	18.3 ± 8.6	18	23.0 ± 12.3	21.2 ± 9.9	−2.14 [−4.1, −0.1]	.037*
Walking Adaptability Ladder Test—double run (s)	16^A^	38.7 ± 17.9	35.1 ± 14.9	18	38.7 ± 16.8	36.3 ± 14.9	−1.26 [−4.3, 1.8]	.412
Time spent active (% of day)	17^B^	9.9 ± 3.4	10.2 ± 2.9	17^B^	10.6 ± 3.0	9.7 ± 2.9	0.52 [−0.9, 1.9]	.447
Time spent walking (% of day)	17^B^	8.1 ± 3.2	8.3 ± 3.0	17^B^	8.2 ± 2.8	7.5 ± 1.6	0.62 [−0.5, 1.8]	.282
Three-dimensional gait analysis#								
Average stride length (m)	18	1.19 ± 0.2	1.24 ± 0.2	18	1.17 ± 0.2	1.19 ± 0.2	0.03 [−0.0, 0.1]	.232
Average stride time (s)	18	1.15 ± 0.16	1.12 ± 0.1	18	1.15 ± 0.2	1.13 ± 0.2	<0.01 [−0.1, 0.1]	.968
Average step width (m)	18	0.16 ± 0.04	0.16 ± 0.04	18	0.15 ± 0.05	0.15 ± 0.05	0.01 [0.0, 0.0]	.116
Cadence (steps/min)	18	106.2 ± 12.0	107.8 ± 10.0	18	106.9 ± 15	109.1 ± 16	<0.01 [0.0, 0.0]	.947
Walking velocity (m/s)	18	1.06 ± 0.3	1.11 ± 0.2	18	1.05 ± 0.2	1.09 ± 0.3	0.02 [0.0, 0.0]	.119

Abbreviations: n, number of participants included in the analysis; SD, standard deviation; CI, Confidence Interval; ANCOVA, analysis of covariance.

Values displayed: unadjusted means ± SD. Results of a 1-way ANCOVA for group differences with pre-intervention assessment (wk 0) as covariate; mean differences represent the differences between the estimated means post-intervention (wk 6) and estimated means pre-intervention (week 0).

Some participants were excluded from the analysis due to missing data resulting from: ^A^an inability to perform the test or ^B^technical issues.

* Indicates significant differences.

# Collected during 2 trials of 3 min overground walking.

### Time Effects

**Figure 2.**
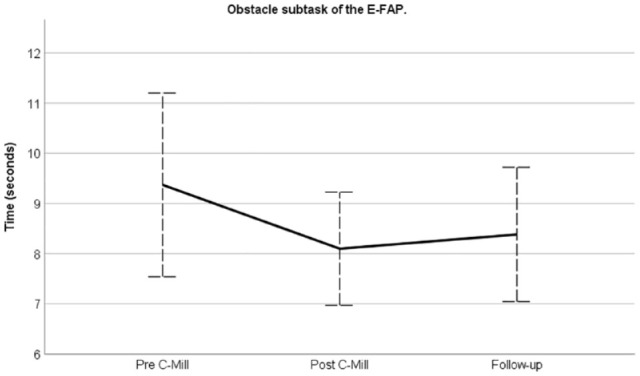
Time effects of C-Mill training on the obstacle subtask for both groups together. Values displayed are unadjusted means. Error bars represent 95% confidence intervals.

Directly following 5 weeks of gait-adaptability training, the participants of both groups reduced the required time to perform the obstacle subtask (mean difference: −1.3 seconds; 95% CI: −2.1, −0.4; *P* < .01) and retained this gain up to the 15-week follow-up assessment (Figure 2). Similar results were found for most secondary outcomes, including the MiniBEST, ABC scale, 10 mWT (comfortable and fast gait speed), WALT single and double run, levels of daily activity, and average stride length and walking velocity recorded during gait analysis (Table 3). The scores of all assessments for each group separately are included in the Supplemental Materials—Table 4 (gait-adaptability training group) and Table 5 (waiting-list control group).

**Table 3.** Group Means of Both Groups Combined (n = 36) Pre Training, Post Training and at Follow-Up and Mean Differences Between Assessments.

	Pre training (n = 36)	Post training (n = 36)	Follow-up (n = 36)	Time effect	Mean group difference between post training and pre training assessment [95% CI]	Mean group difference between follow-up and post training assessment [95% CI]
	Mean ± SD	Mean ± SD	Mean ± SD	*P* value		
Clinical assessment						
Obstacle subtask (s)	9.4 ± 5.4	8.1 ± 3.3	8.4 ± 4.0	.006	−1.3 [−2.1, −0.4]**	0.3 [−0.1, 0.6]
MiniBEST	19.1 ± 5.0	21.6 ± 4.6	21.2 ± 5.2	<.001	2.5 [1.7, 3.3]**	−0.4 [−1.0, 0.3]
Activities-specific balance confidence scale (%)	69.6 ± 17.4	74.1 ± 16.4	73.1 ± 15.3	.010	4.5 [1.3, 7.7]**	−1.0 [−3.7, 1.5]
Ten-meters walk test—comfortabel (m/s)	1.3 ± 0.3	1.3 ± 0.3	1.3 ± 0.2	.001	0.1 [0.0, 0.1]**	0.0 [−0.1, 0.0]
Ten-meters walk test—fast (m/s)	1.7 ± 0.3	1.7 ± 0.3	1.7 ± 0.3	.003	0.1 [0.0, 0.1]**	0.0 [−0.1, 0.0]*
Walking adaptability ladder test—single run (s)	21.5 ± 11.7	18.4 ± 7.9	19.2 ± 10.2	<.001	−3.1 [−4.6, 1.5]**	0.8 [−3.4, 1.7]
Walking adaptability ladder test—double run (s)	37.4 ± 16.2	34.0 ± 14.7	33.9 ± 14.6	<.001	−3.4 [−4.9, −1.9]**	−0.1 [−1.7, 1.4]
Time spent active (% of day)	9.8 ± 3.1	10.3 ± 3.1	9.9 ± 3.5	.709	0.2 [−0.4, 0.9]	−0.4 [−1.2, 0.3]
Time spent walking (% of day)	7.8 ± 2.5	8.3 ± 3.0	7.4 ± 2.9	.078	0.2 [−0.4, 0.9]	−0.9 [−1.6, −0.2]
Three-dimensional gait analysis^#^						
Average stride length (m)	1.2 ± 0.2	1.3 ± 0.2	1.2 ± 0.2	<.001	0.1 [0.0, 0.1]**	−0.0 [−0.0, −0.0]*
Average stride time (s)	1.1 ± 0.2	1.1 ± 0.1	1.1 ± 0.1	.273	0.0 [0.1, 0.0]	0.0 [0.0, 0.0]
Average step width (m)	0.15 ± 0.0	0.15 ± 0.0	0.16 ± 0.0	.372	0.0 [0.0, 0.0]	0.0 [0.0, 0.0]
Walking velocity (m/s)	1.1 ± 0.2	1.1 ± 0.2	1.1 ± 0.2	.001	0.1 [0.0, 0.1]**	−0.0 [−0.1, 0.0]*
Cadence (steps/min)	107.6 ± 13.8	109.0 ± 11.8	108.6 ± 11.1	.417	1.4 [−1.0, 3.8]	−0.4 [−2.0, 1.3]

Values displayed are unadjusted means ± SD. Presented data includes the pre-training, post-training and follow-up assessment data for the gait-adaptability training group and waiting-list control group (gait-adaptability training group: assessments 1, 2, 3; waiting-list control group: assessments 2, 3, 4).

* Indicates significant differences at *P* < .05.

** Indicates significant differences at *P* < .01.

# Collected during 2 trials of 3 min overground walking.

### Fall Diaries

In the 15 weeks prior to the first assessment, 26 falls were reported among 12 out of 34 participants (35%; 1-4 falls per participant). In the 15-week following the gait-adaptability training, 26 falls were reported among 12 out of 34 participants (35%; 1-5 falls per participant).

In the 15 weeks prior to the first assessment, 291 near-falls were reported among 22 out of 34 participants (65%; 1-75 near-falls per participant). In the 15 weeks following the gait-adaptability training, 200 near-falls were reported among 11 out of 34 participants (32%; 1-63 near-falls per participant). Nineteen participants reported a decrease in their near-falls (56%; 1-42 near-falls per participant), whereas 2 participants reported an increase of 1 and 33 near-falls, respectively. Additionally, 2 participants reported an identical count of near-falls.

## Discussion

Move-HSP is the first randomized clinical trial investigating the effects of gait-adaptability training in people with pure HSP. Our results showed that, following gait-adaptability training, participants improved on the obstacle subtask of the E-FAP as well as on various secondary outcome measures of balance and gait. However, our results did not confirm the hypothesis that adding gait-adaptability training to usual care would result in greater improvements on these outcome measures.

Previous uncontrolled studies using a pre-post assessment design reported that gait, balance, and/or gait adaptability performance improved following 3.5 to 10 hours of C-Mill training in people with Parkinson’s disease,^13^ stroke,^9^ and cerebellar degeneration.^12^ In addition, a previous randomized controlled trial in people with chronic stroke reported that the primary outcome walking speed did not show a greater improvement following C-Mill training compared to overground gait adaptability training. Yet, additionally, they reported that the C-Mill training group did show a greater improvement on context-specific walking speed (secondary outcome) directly post intervention, but this effect was not retained after 5 weeks follow-up. The results of the current study are coherent with the above-mentioned studies, as across both groups, the obstacle subtask of the E-FAP showed a significant improvement of 1.3 seconds directly post intervention, that was retained after 15 weeks follow-up. However, 5 weeks of gait-adaptability training added to usual care did not lead to a greater improvement of gait adaptability compared to usual care alone. Of note, we did find a greater improvement on the single run of the WALT—a novel test designed to evaluate walking adaptability^21^—in the gait-adaptability training group compared to the waiting-list control group. The potential utility of the WALT to evaluate gait adaptability should be investigated in future trials.

Surprisingly, during the waiting-list period, participants in the control group improved on the obstacle subtask of the E-FAP as well as on most of the secondary outcomes, including measures of balance, balance confidence, gait speed, and gait adaptability. These improvements in the control group may be explained in 2 ways. First, they may have been influenced by the so-called Hawthorne effect,^23^ the potential change in behavior that occurs when people become aware of being observed and examined.^24-27^ A second explanation relates to the impact of the Covid-19 pandemic. Unfortunately, we had to postpone the first assessments in all participants until the lockdown related to the Covid-19 restrictions was over. Previous research from our group showed that, during the Covid-19 pandemic, people with HSP were generally less active,^28^ a phenomenon that has also been reported in other neurological populations.^29-34^ In addition, we found that the relative inactivity during the Covid-19 lockdown negatively impacted on spasticity-related symptoms, including gait and balance impairments. Because—in the current study—the first assessments took place shortly after a period of generally reduced levels of physical activity, both groups may have increased their levels of activity and exercise in such a way that the added effects of gait-adaptability training were reduced.

This study has several strengths and limitations. Despite a delay of 4 months due to the Covid-19 pandemic, we were able to conduct the trial according to the previously published protocol.^14^ We were able to recruit the required number of participants and had no participant drop-out during the trial. Moreover, the adherence to the gait-adaptability training was very high. The generalizability of our results to the population of ambulatory people with HSP at large is expected to be high as well, as our participants showed large clinical heterogeneity (i.e., disease severity, disease duration, and muscle tone), the use of orthotics was allowed, and the level of independent ambulation varied between the ability to walk 50 m and completion of a marathon (i.e., 42 km). We cannot fully rule out a ceiling effect in the E-FAP obstacle subtask results (limiting room for improvement), however comparison of the current HSP data with unpublished control data obtained by us in healthy controls of similar age (N = 15; 49.0 ± 11.5 years) indicated that only 6 of our 36 HSP participants completed the E-FAP obstacle subtask within a normal range (mean + 2SD of healthy controls).

The most important shortcoming of this study was that assessors were not blinded for group allocation. To limit observational bias, a standardized protocol was used for all assessments. In addition, the current protocol implemented a relatively short training period of 5 weeks. Although this is in line with previous studies,^9,12^ it remains unknown if people with HSP would benefit from a longer training period, including the use of booster sessions. Furthermore, although we did include the ABC—a self-perceived balance confidence scale—we lack qualitative feedback from our participants on how they perceived the gait-adaptability training. Lastly, we have no details about the content of the usual care (e.g., to what extent aspects of gait adaptability were trained). Hence, it is possible that the contrast between the gait-adaptability training group and the usual care control group was insufficient.

With regard to clinical implications, our study provides insufficient evidence to conclude that 5 weeks of gait-adaptability training, added to usual care, leads to greater improvement of gait-adaptability performance in people with HSP compared to usual care alone. Our study has several implications for future research. Future studies should focus on the evaluation of long-term gait-adaptability training, including booster sessions. They should also make use of a validated outcome measure that is more sensitive than the E-FAP obstacle subtask to the acquisition of complex gait and dynamic balance skills. Lastly, future studies should include qualitative assessments of the intervention by patients to improve its feasibility, content and attractiveness.

## Supplemental Material

sj-docx-1-nnr-10.1177_15459683221147839 – Supplemental material for Gait-Adaptability Training in People With Hereditary Spastic Paraplegia: A Randomized Clinical Trial Click here for additional data file. Supplemental material, sj-docx-1-nnr-10.1177_15459683221147839 for Gait-Adaptability Training in People With Hereditary Spastic Paraplegia: A Randomized Clinical Trial by Lotte van de Venis, Bart van de Warrenburg, Vivian Weerdesteyn, Alexander C. H. Geurts and Jorik Nonnekes in Neurorehabilitation and Neural Repair
