# Supplementary material for: Gait-Adaptability Training in People With Hereditary Spastic Paraplegia: A Randomized Clinical Trial
Source: Neurorehabil Neural Repair. 2023 Jan 25;37(1):27–36. doi: 10.1177/15459683221147839 (PMC9896539; doi:10.1177/15459683221147839)
Supplement: sj-docx-1-nnr-10.1177_15459683221147839 – Supplemental material for Gait-Adaptability Training in People With Hereditary Spastic Paraplegia: A Randomized Clinical Trial [file sj-docx-1-nnr-10.1177_15459683221147839.docx]

**Supplementary materials**

Table 4. Group means of the gait-adaptability training group (n=18) pre training, post training and at follow-up.

|  |  | **Pre training** | **Post training** | **Follow-up** |
| --- | --- | --- | --- | --- |
|  |  | Mean ± SD | Mean ± SD | Mean ± SD |
| **Clinical Assessment** |  |  |  |  |
| Obstacle subtask (s) |  | 10.3 ± 6.6 | 8.6 ± 3.9 | 8.8 ± 4.5 |
| MiniBEST |  | 18.3 ± 6.0 | 20.1 ± 5.4 | 20.0 ± 5.9 |
| Activities-specific balance confidence scale (%) |  | 66.5 ± 18.1 | 72.7 ± 16.2 | 72.2 ± 13.4 |
| Ten-meters walk test – Comfortabel (m/s) |  | 1.3 ± 0.3 | 1.3 ± 0.3 | 1.3 ± 0.2 |
| Ten-meters walk test – Fast (m/s) |  | 1.6 ± 0.3 | 1.7 ± 0.4 | 1.7 ± 0.3 |
| Walking adaptability ladder test – single run (s) |  | 21.6 ± 13.8 | 18.3 ± 8.6 | 19.4 ± 11.5 |
| Walking adaptability ladder test – double run (s) |  | 38.7 ± 17.9 | 35.1 ± 14.9 | 33.6 ± 13.7 |
| Time spent active (% of day) |  | 9.8 ± 3.4 | 10.2 ± 2.9 | 9.5 ± 3.7 |
| Time spent walking (% of day) |  | 8.1 ± 3.0 | 8.3 ± 3.0 | 7.4 ± 3.6 |
| **Three-dimensional gait analysis ^#^** |  |  |  |  |
| Average stride length (m) |  | 1.2 ± 0.2 | 1.2 ± 0.2 | 1.2 ± 0.1 |
| Average stride time (s) |  | 1.1 ± 0.2 | 1.1 ± 0.1 | 1.1 ± 0.1 |
| Average step width (m) |  | 0.2 ± 0.0 | 0.2 ± 0.0 | 0.2 ± 0.0 |
| Walking velocity (m/s) |  | 1.1 ± 0.3 | 1.1 ± 0.2 | 1.1 ± 0.2 |
| Cadence (steps/min) |  | 106.2 ± 11.9 | 107.8 ± 10.0 | 107.8 ± 9.0 |

Values displayed are means ± standard deviation. Presented data includes the pre-training, post-training and follow-up assessment data for
the gait-adaptability training group. ^#^ Collected during two trials of 3 minute overground walking.

Table 5. Group means of the waiting-list control group (n=18) pre waiting-list, pre training, post training and at follow-up

|  |  | **Pre waiting-list** | **Pre training** | **Post training** | **Follow-up** |
| --- | --- | --- | --- | --- | --- |
|  |  | Mean ± SD | Mean ± SD | Mean ± SD | Mean ± SD |
| **Clinical Assessment** |  |  |  |  |  |
| Obstacle subtask (s) |  | 9.5 ± 4.7 | 8.5 ± .3.8 | 7.6 ± 2.7 | 7.9 ± 3.3 |
| MiniBEST |  | 19.3 ± 3.8 | 19.9 ± 3.8 | 23.1 ± 3.1 | 22.5 ± 9.1 |
| Activities-specific balance confidence scale (%) |  | 70.9 ± 18.2 | 72.7 ± 16.6 | 75.5 ± 16.8 | 74.0 ± 17.3 |
| Ten-meters walk test – Comfortabel (m/s) |  | 1.2 ± 0.3 | 1.3 ± 0.3 | 1.4 ± 0.3 | 1.3 ± 0.3 |
| Ten-meters walk test – Fast (m/s) |  | 1.7 ± 0.4 | 1.7 ± 0.3 | 1.8 ± 0.3 | 1.7 ± 0.3 |
| Walking adaptability ladder test – single run (s) |  | 23.0 ± 12.3 | 21.2 ± 9.9 | 18.6 ± 7.6 | 19.0 ± 9.1 |
| Walking adaptability ladder test – double run (s) |  | 38.7 ± 16.8 | 36.3 ± 14.6 | 33.1 ± 14.9 | 34.1 ± 15.3 |
| Time spent active (% of day) |  | 10.6 ± 3.0 | 9.7 ± 2.8 | 10.5 ± 3.4 | 10.3 ± 3.4 |
| Time spent walking (% of day) |  | 8.2 ± 2.8 | 7.5 ± 1.6 | 8.2 ± 3.1 | 7.3 ± 2.0 |
| **Three-dimensional gait analysis ^#^** |  |  |  |  |  |
| Average stride length (m) |  | 1.2 ± 0.2 | 1.2 ± 0.2 | 1.3 ± 0.2 | 1.2 ± 0.2 |
| Average stride time (s) |  | 1.2 ± 0.2 | 1.1 ± 0.2 | 1.1 ± 0.2 | 1.1 ± 0.2 |
| Average step width (m) |  | 0.2 ± 0.2 | 0.2 ± 0.1 | 0.2 ± 0.1 | 0.1 ± 0.1 |
| Walking velocity (m/s) |  | 1.0 ± 0.2 | 1.1 ± 0.3 | 1.2 ± 0.3 | 1.1 0.2 |
| Cadence (steps/min) |  | 107.0 ± 15.4 | 109.1 ± 15.6 | 110.2 ± 0.3 | 109.5 ± 13.1 |

Values displayed are means ± standard deviation. Presented data includes the pre waiting-list, pre training, post training and follow-up assessment data
for the waiting-list control group. ^#^ Collected during two trials of 3 minute overground walking.
